# Artificial intelligence and thyroid disease management: considerations for thyroid function tests

**DOI:** 10.11613/BM.2022.020601

**Published:** 2022-06-15

**Authors:** Damien Gruson, Pradeep Dabla, Sanja Stankovic, Evgenija Homsak, Bernard Gouget, Sergio Bernardini, Benoit Macq

**Affiliations:** 1 Department of Clinical Biochemistry, Cliniques Universitaires Saint-Luc, Université catholique de Louvain, Ottignies-Louvain-la-Neuve, Belgium; 2 Department of Biochemistry, Pant Institute of Postgraduate Medical Education & Research, Delhi, India; 3 Center for Medical Biochemistry, University Clinical Center of Serbia, Beograd, Serbia; 4 Department for Laboratory Diagnostics, University Clinical Center Maribor, Maribor, Slovenia; 5 Healthcare Division Committee, Comité Français d’accréditation, Paris, France; 6 Department of Experimental Medicine, University of Tor Vergata, Rome, Italy; 7 Institute of Information and Communication Technologies, UCLouvain, Ottignies-Louvain-la-Neuve, Belgium

**Keywords:** artificial intelligence, biomarkers, clinical decision support system, digital health, thyroid diseases

## Abstract

Artificial intelligence (AI) is transforming healthcare and offers new tools in clinical research, personalized medicine, and medical diagnostics. Thyroid function tests represent an important asset for physicians in the diagnosis and monitoring of pathologies. Artificial intelligence tools can clearly assist physicians and specialists in laboratory medicine to optimize test prescription, tests interpretation, decision making, process optimization, and assay design. Our article is reviewing several of these aspects. As thyroid AI models rely on large data sets, which often requires distributed learning from multi-center contributions, this article also briefly discusses this issue.

## Introduction

Artificial intelligence (AI) is a field of computing science mimicking the human thought processes and behaviours used to make decisions or take actions (*1*). It uses different mathematical and algorithmic approaches, from operational research to constrained programming (*1*).

Artificial intelligence is transforming healthcare and offers new promising solutions in clinical examination, precision medicine, research, and clinical diagnostics (*1*-*4*). The expectations associated to AI are growing exponentially as the volume of medical data available (electronic medical records, laboratory informatics systems, omics, mobile health applications, *etc*.) is constantly increasing (*5*). In the field of laboratory medicine, automation and digitalization are stimulating the use of AI and the evolution of laboratory services (*2*, *3*). Artificial intelligence also allows disorders and outcome forecast from routine laboratory analysis and understanding of complex biochemical information (*6*). The adoption of AI has been accelerated by the COVID-19 pandemic and AI has effectively demonstrated to assume a significant part in checking COVID-19 through extending the spread of infection, contact following, early recognition, observing, social removing, incorporating information and preparing of medical workers (*7*).

Thyroid hormones are fundamental for development, neuronal growth, fertility, and metabolism (*8*). Thyroid diseases are frequent conditions, affecting millions of people around the world, related to multiple health problems and for which thyroid function tests (TFT) are frequently ordered for diagnosis and monitoring of diseases (*9*).

We aim with this short article to review some of the potential applications of AI on thyroid function tests.

## The application of AI to thyroid disorders: preliminary observations from radiology and imaging

The complexity of the diagnosis of some thyroid pathologies had stimulated the development of AI solutions to assist physicians. Different examples coming from the field of radiology and imaging can illustrate this trend.

A first observation is that using AI based computerized diagnosis systems can personalize and optimize the management of thyroid nodules. Because of clinical requests to diminish superfluous fine needle aspiration (FNA), AI-based solutions have been proposed as ways of expanding the exactness of ultrasonography-based conclusion for less-experienced administrators and to address the intricacy of the fragmented risk stratification systems (*10*).

A second observation is the possibility for staging malignancy of thyroid tumors and how deep-learning AI model can help to differentiate between malignant tumors and benign thyroid tumors based on distinctive clinical and ultrasonographic characteristics (*11*). Artificial intelligence algorithms have been utilized for the classification of thyroid nodules utilizing ultrasound pictures, cytopathology pictures, and molecular markers (*12*). Interestingly, the accuracy of the AI model was superior to that of radiologists for diagnostic performances of malignancy and was leading to significant reduction of FNA (*10*). Published data showed that neural network precision in segregating advanced *versus* non-advanced thyroid carcinomas was 84%, with positive and negative predictive values of 87% and 92%, respectively (*13*). The use of AI to determine tumor classification represents also an important step for the choice of an optimum treatment (*11*).

A third observation is the application of AI for the automation of image analysis with whole-slide imaging (*14*). A recent study reported that the coefficients of correlation with manual evaluation were higher than 0.76 and that the diagnosis performance of the AI based robotized models was similar to a specialist pathologist analysis (*14*).

## The application of AI to laboratory medicine: considerations for thyroid function tests

**Figure 1:**
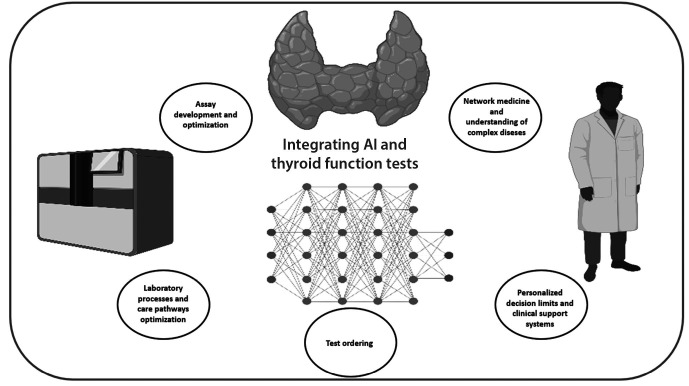
The perspectives associated to the integration of AI and thyroid function tests. The figure was prepared with the use of www.biorender.com. AI - Artificial intelligence.

Thyroid function tests represent an important asset for physicians in the diagnosis and monitoring of thyroid pathologies. Artificial intelligence applications have the potential to optimize correct test prescription, tests interpretation, decision making, process optimization, and assay desing (Figure 1). We will discuss some of these perspectives at the preanalytical, analytical and post-analytical levels.

### Impact on preanalytical factor and process optimization

An important aspect within the preanalytical phase is the appropriate test ordering by physicians. Guiding the physicians for the ordering of the right TFT according to clinical context is important, especially in a context where the over-use of TFT has been documented in both hospital and primary care practices (*15*). The optimal choice of TFT is important to make effective clinical decision, to help physicians spend more time treating patients (*16*). In contrast, excessive TFT ordering policies can prompt financial weight in a period of rising medical care costs (*8*). Artificial intelligence based companions have the potential to help physicians to optimize TFT prescription and to define intelligent order sets that can contribute to reduce laboratory overutilization (*15*). A recent study investigated the value of deep learning-based automated system to recommend appropriate laboratory tests (*16*). The AI based model achieved a higher area under the receiver operating characteristics curve (AUROC micro = 0.98, and AUROC macro = 0.94). The integration of such AI companion into existing workflows can reduce under- and over-utilization of TFT (*17*).

Clinical laboratories are still in important phases of consolidation and automation, with emphasis placed also at the preanalytical levels and samples transportation. Clinical laboratories also constantly work on the improvement of patients’ experience and laboratory services. Artificial intelligence can possibly upgrade all degrees of testing work process and tests assortment, including work process enhancement and operational efficiency (*18*, *19*).

Artificial intelligence tools can also have application to assist medical teams for matrix selection at the time of tube collection, recommendations of adequate moment for sampling and integration of variables to control and monitor transportation of samples.

### Impact on the analytical phase

Improvement of TFT assays remain an objective for the clinical community and for scientific societies (*20*). The standardization is also still ongoing with the perspective of better commutability of results and clinical cut-off points between laboratories (*20*). However, several challenges are still paving this way and the use of AI for the *in silico* design of TFT assays can offer an additional solution to go forward (*21*). *In silico* modelling can enhance the know-how on TFT assays and technology capabilities with perspectives of increased efficiency and robustness of assays, reduction of time-to-market and streamline of operations and production. *In silico* approaches can be applied to immunoassays for epitope prediction, simulation of optimized assay sequences and formats and validation of novel proof of concept at the bioprocessing level.

Additionally, as TFT are still prone to interferences with consequences for patients as up to half of recorded thyroid obstructions prompted misdiagnosis or potentially unseemly administration, including remedy of a superfluous treatment, the *in silico* design can significantly decrease the sensitivity to interfering compounds and improve TFT assays (*22*).

### Impact on post-analytical phase

Definition of personalized reference intervals is important for the appropriate use of TFT. The use of data mining and AI approaches can help clinical laboratory teams for the establishment or verification of reference intervals for TFT by extracting and integrating data from laboratory informatic systems and electronic medical records (*23*). Large amount of data can be processed and reference intervals more accurately adjusted to different population subgroups.

Thyroid function tests variability within and between subjects is high with the example of TSH concentrations which can change over the long run inside a person because of different internal and outer elements (*24*). Artificial intelligence tools could be seen also as additional options to better estimate and derive biological variability by diving into clinical databases to identify sources of fluctuations for TFT and therefore adjust decision limit accordingly (*25*). Recent data showed that AI and machine learning methods might offer unique insight into the complex hypothalamic-pituitary-thyroid axis, identifying factors determining individual TSH concentration, and may be relevant tool that guides us in making appropriate therapeutic decisions for the individual patient (*26*).

### Clinical decision support systems

Clinical decision support systems (CDSS) are designed to utilize medical data, knowledge, and analysis engines to generate patient-specific assessments or recommendations to health professionals in order to assist decision making (*27*). Artificial intelligence can enable CDSS that aid the decision-making process for thyroid diseases through an intelligent component. Building of CDSS for the interpretation of TFT and image signals will provide integrated approaches to support clinical decisions regarding thyroid diseases and the integration of clinical practice guidelines. Clinical decision support systems using machine learning-evaluated geometric and morphological features have already been evaluated for the classification of thyroid nodules (*28*). The COVID-19 pandemic also triggers the development of CDSS using imaging techniques and biomarkers for mortality prediction (*29*). Same strategy could be applied to thyroid cancer using the progresses of AI to separate and break down morphological, textural, and molecular features (*30*). Clinical decision support systems might help physicians to accelerate the decision-making process in thyroid diseases. Another example is the potential use of AI based CDSS for diagnosis and estimating the risk for development of thyroid autoimmune disease (*31*). Using AI based CDSS to potential autoimmune thyroid pathobiology will rely on the integration of complex datasets coming from genetic (human leukocyte antigen (HLA) and other genes), environmental (different triggers (viruses, microbes)) and immune system characteristics (autoantibodies, cytokines) (*32*, *33*). This could be particularly relevant to distinguish different types of thyroid autoimmune diseases such as autoimmune thyroiditis, Hashimoto thyroiditis, Hashimoto disease or Grave disease with different patterns of hyperthyroidism or hypothyroidism (*32*, *33*). The application of AI in autoimmune diseases in most recent studies are focused on patient identification, hazard expectation, finding, disorder subtype and evolution as well as complications (*31*). Diagnosis solution based on artificial immune recognition system with balanced pre-processing is one of the promising future method for evaluation thyroid diseases (*32*).

## Challenges and additional perspectives

### Challenges

In order to achieve the clinical benefits of using AI for thyroid pathology and for optimized use of TFT, several challenges must be considered (*6*, *34*):

- Establish a comprehensive legal framework for AI and update existing relevant legislations to ensure that they are fit for purpose.
- Identify and promote best practices ensuring the robustness of big data and AI systems in the health sector both at the stages of development and actual use to reduce potential biases and errors of AI-based decision making.
- Improve data interoperability and support the development of data infrastructure, with the goal of providing a reliable flow data with standardized formats, the necessary cybersecurity provisions, and data protections.
- Support the development of national electronic health records and improve the interoperability of health data.
- Stimulate scientific research and development in the field to boost the updates of AI applications to healthcare and support patient access to the best available technologies.
- Equip the workforce with the necessary skill sets to maximize the positive impact of AI and conduct a comprehensive regulatory assessment of the medical professions frameworks to determine whether they are fit for the use of patient-centered AI in healthcare provision.
- Ensure that AI is applied in full respect of data protection rules while observing the balance between scientific advancement and patient protection.
- Put in place mechanisms to ensure educational assistance to patients to allow them to better understand and use AI and empower them to actively participate in the management of their health.
- Define the value and business models around AI tools and carefully assess the benefits in terms of clinical outcomes, patient experience, and costs.
- Encourage the active involvement of healthcare workforces for the construction and validation of AI solutions and decision support systems.

### Continuous learning in secure multi-centric coalitions

Although recent research results have shown effective and promising results obtained by AI models for diagnosis, classification, and clinical decision support for the treatment of thyroidal disorders, the deployment of these models must adapt to the variability of data acquisition across the clinical environments. Therefore, the use of continual learning models which adapt to local clinical practices must be triggered. Such models should be promoted inside coalitions of hospitals sharing the same practices and guidelines. A solution to provide efficient, reliable and privacy-preserving distributed learning inside a coalition of hospitals has been previously described (*35*).

### Network medicine

Thyroid pathology encompasses a heterogeneous group of clinic-pathological entities (*30*). Network medicine offers the possibility to integrate multi-omics data with very well characterized clinical phenotype to work on the comprehension of complex diseases (*36*). Using AI to apply network medicine to thyroid pathobiology will rely on the integration of complex datasets coming from hyperspectral mass spectrometry imaging, from the molecular signatures of thyroid tumors, from proteomic analysis and from transcriptome or other multi-omics networks (*37*-*40*). The combination of AI and network medicine will partake to a superior comprehension of thyroid disorders and to the improvement of upgraded diagnostic and treatment options by elicitation of causal relationships in the biological continuum, from molecular omics data, through histology and to (*40*).

## Conclusion

Artificial intelligence is becoming a useful tool to assist diagnosis and risk classification in thyroid diseases. Artificial intelligence companions can significantly improve the performances of clinical laboratories for process optimization as well as providing support for clinical decision. Artificial intelligence tools offer to the *in vitro* diagnostic companies’ novel options for the design and improvement of TFT. The incorporation of AI into clinical pathways also offers opportunity to enhance care pathways, laboratory tests prescription, clinical diagnosis, and patients’ outcomes. In the context of network medicine, AI will help to better understand complex thyroid diseases, detect molecular mechanisms and potential new treatment strategies.
